# Predicting Interpersonal Outcomes From Information Processing Tasks Using Personally Relevant and Generic Stimuli: A Methodology Study

**DOI:** 10.3389/fpsyg.2020.543596

**Published:** 2020-09-24

**Authors:** Lisa Serravalle, Virginia Tsekova, Mark A. Ellenbogen

**Affiliations:** Centre for Research in Human Development, Concordia University, Montréal, QC, Canada

**Keywords:** information processing, personally relevant stimuli, interpersonal outcomes, emotion, attentional biases

## Abstract

Despite evidence of differential processing of personally relevant stimuli (PR), most studies investigating attentional biases in processing emotional content use generic stimuli. We sought to examine differences in the processing of PR, relative to generic, stimuli across information processing tasks and to validate their use in predicting concurrent interpersonal functioning. Fifty participants (25 female) viewed generic and PR (i.e., their intimate partner’s face) emotional stimuli during tasks assessing selective attention (using a modified version of the Spatial Cueing Task) and inhibition (using the Negative Affective Priming task) of emotional content. Ratings of relationship quality were also collected. Evidence of increased selective attention during controlled and greater avoidance during automatic stages of processing emerged when viewing PR, relative to generic, emotional faces. We also found greater inhibition of PR sad faces. Finally, male, but not female, participants who displayed greater difficulty disengaging from the sad face of their partner reported more conflict in their relationships. Taken together, findings from information processing studies using generic emotional stimuli may not be representative of how we process PR stimuli in naturalistic settings.

## Introduction

Facial expressions of emotion are salient and evolutionary-significant visual stimuli when engaging in social interactions (Keltner and Gross, 1999). In fact, the accurate recognition of emotions in facial expressions is integral to meaningful social interactions by allowing individuals to infer the emotional states of others and to anticipate future responses (Russell et al., 2003). On the other hand, the presence of attentional biases or deficits in facial emotion processing is associated with high levels of anxiety (Mathews and MacLeod, 2005; Bar-Haim et al., 2007) and depressive symptoms (Gotlib et al., 2004; Ellenbogen and Schwartzman, 2009). For example, individuals with high trait anxiety or a diagnosis of an anxiety disorder are slower at disengaging their attention away from masked angry faces (presented with limited conscious awareness), relative to those with low trait anxiety or healthy controls, respectively (Fox et al., 2002; Ellenbogen and Schwartzman, 2009). Similarly, individuals with depression are slower to shift their attention from sad faces relative to controls and individuals with anxiety disorders (Gotlib et al., 2004; Ellenbogen and Schwartzman, 2009). In the above studies and the vast majority of research in this area (Mogg and Bradley, 1998; Hallion and Ruscio, 2011; Heeren et al., 2015), the experimental protocols used stimuli of emotional faces from validated databases, which are typically derived from trained actors portraying the extreme depiction of each emotion. Other studies use words associated with an emotional state, such as “death” or “happy.” Although these “generic” emotional stimuli are useful in identifying general attentional biases and the brain circuits associated with different emotions (Lang et al., 2000), this methodology may be limited in its generalizability to naturalistic situations. Indeed, a growing body of literature suggests that generic stimuli may be processed differently than stimuli that are meaningful and relevant to the individual (Bartels and Zeki, 2000; Leibenluft et al., 2004; Wingenfeld et al., 2006; Claypool et al., 2007; Gobbini and Haxby, 2007; Fossati, 2012; Fridrici et al., 2013). In one study using the emotional Stroop task, participants displayed greater interference in reaction times when viewing personally relevant words, relative to generic negative or neutral words (Wingenfeld et al., 2006). Individuals with comorbid borderline personality disorder and post-traumatic stress disorder were slower to name the color of negative personally relevant words, relative to generic negative and neutral words (Wingenfeld et al., 2009). A related body of research has documented similar differences in the processing of self-vs. other-related stimuli (Ibáñez et al., 2011; Salehinejad et al., in press). For example, persons with elevated depressive symptoms, relative to those with no symptoms, exhibited reduced attention to positive words when the words were processed as being self-referential, but not when the words were meant to describe another person (Ji et al., 2017).

With regards to processing emotion in faces, researchers have shown that that individuals may perceive facial expressions of familiar (i.e., faces which individuals have been previously exposed to) and unfamiliar faces differently. For example, participants perceived familiar faces as expressing more happiness than unfamiliar faces even when the facial expression of happiness was identical (Claypool et al., 2007). Furthermore, enhancing familiarity through mere exposure to stimuli might alter selective attention during computer tasks. In one study, individuals attended less to threatening and more to neutral stimuli following mere exposure, relative to novel stimuli of equal emotional valence (Young and Claypool, 2010). These findings are not surprising, given the existing research suggesting different neural networks are involved in the recognition of familiar and unfamiliar faces, and in particular the amygdala and the insula, two brain structures implicated in the processing of emotional stimuli (Gobbini and Haxby, 2007; Fossati, 2012).

Indeed, differential neural responding to familiar and unfamiliar stimuli is of great importance to social neuroscience research. For example, there is evidence of different neural activation, mostly in brain areas that mediate emotional responses (amygdala, medial insula, anterior paracingulate cortex, and posterior superior temporal sulcus), when processing the face of a romantic partner vs. that of a close friend (Bartels and Zeki, 2000), and between the way a mother processes the face of her own child vs. that of a familiar but unrelated child (Leibenluft et al., 2004). Researchers have also found greater activation in autobiographical memory circuits when viewing familiar stimuli. Specifically, Viskontas et al. (2009) found increased activation of the medial temporal lobe when viewing photographs of individuals with whom they had previous contact, such as family members and the experimenter, compared to familiar but not necessarily personally relevant (e.g., celebrities) and unfamiliar stimuli. The association between familiarity of visual stimuli and increased medial temporal lobe activation has been replicated across various studies (Elfgren et al., 2006; Weibert et al., 2016). These findings may be reflective of the greater number of memories and experiences attached to personally relevant stimuli, independent of the image itself (Viskontas et al., 2009).

Taken together, both stronger activation of neural circuits associated with emotion processing and brain areas involved in autobiographical memory may, in part, underlie differences in the processing of personally relevant stimuli relative to generic stimuli. Moreover, faces of different types of familiar persons may not be processed uniformly. Viewing one’s intimate partner’s face resulted in higher neural activation in the greatest number of cortical regions, especially in areas associated with emotional processing, than viewing the face of a parent or one’s own face (Taylor et al., 2009). Using event-related potentials and pupillometry, increased attention and arousal were found when participants viewed positive, negative or neutral words in sentences referring to their romantic partner or best friend compared to those referring to an unknown person (Bayer et al., 2017). Despite evidence suggesting substantive differences in how personally relevant stimuli are processed in the brain relative to generic stimuli, especially when viewing the face of a romantic partner (Taylor et al., 2009), personally relevant stimuli have rarely been used in tasks assessing attention and social information processing. Therefore, one goal of the current study was to compare the information processing of emotional faces using generic and personally relevant stimuli.

As a second study aim, we sought to examine whether information processing with personally relevant stimuli predicts interpersonal functioning, in an effort to show evidence of predictive validity. While the use of generic stimuli may be beneficial in studying basic behavioral responses, such as fear (Lundqvist et al., 2004), it is unsure whether these stimuli are useful when studying complex human social behavior, such as interpersonal functioning. Studies using generic stimuli have examined whether selective attention, as a form of emotion regulation, might serve to moderate affect during stressful circumstances. For example, participants who were slow to disengage their attention from supraliminal dysphoric stimuli exhibited a more pronounced negative mood response following an interpersonal stressor (Ellenbogen et al., 2006). Similar findings have linked attentional bias to aversive information with increased reactivity to a stressor (MacLeod et al., 2002). These data suggest that persons with strong attentional bias may have disruptions in their interpersonal functioning given the lack of flexibility to deal effectively with challenging circumstances. However, few studies directly assessed the impact of biased attention on interpersonal relationships. There is some evidence that attentional avoidance of generic fearful, sad and neutral facial expressions mediates the relation between early life stressors and social problems later on (Humphreys et al., 2016; Mastorakos and Scott, 2019). We have reported that the inhibition of personally relevant angry faces was found to moderate the relation between empathy and interpersonal functioning, so that persons with high empathy, but difficulties inhibiting highly relevant and salient stimuli, exhibited poor interpersonal functioning (Iacono et al., 2015). Taken together, comparing the use of generic vs. personally relevant stimuli when examining the link between attentional biases and interpersonal outcomes warrants further exploration.

**FIGURE 1:**
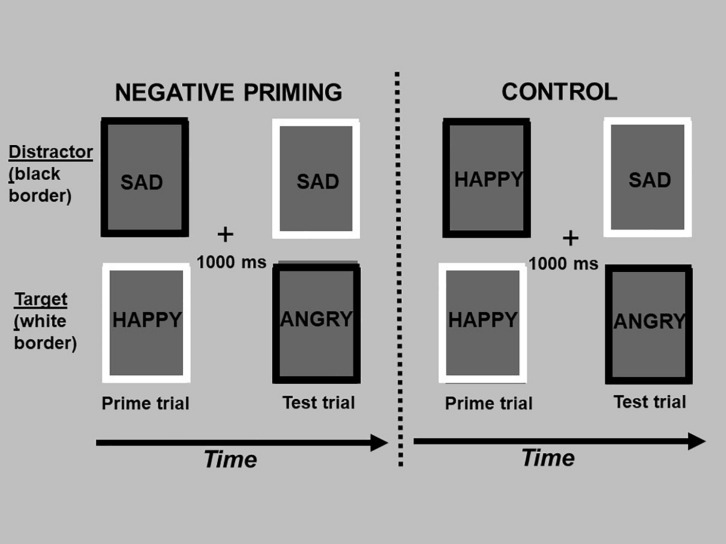
The figure depicts the design of the negative affective priming task, consisting of “prime” and “test” trials. A prime trial (1st and 3rd column) consisted of a target picture with a white border and distractor picture (to be ignored) with a black border, and was always followed by a test trial (2nd and 4th column) 1,000 ms later (“+” fixation between trials). In negative priming test trials, the emotional expression of the target picture (sad face, top picture in the 2nd column) was the same as the emotional expression of the previously ignored picture in the prime trial (top picture in the 1st column). In the control test trials, the emotional expression of the target picture (sad face; top picture in the 4th column) was unrelated to the emotional expression of the previously ignored picture in the prime trial (top picture in the 3rd column). The test trials of the negative priming and control sequences are identical; they only differ in the type of emotion that was ignored in the previous prime trial. The words in each rectangle depict pictures of facial expressions of emotion, which were omitted to limit exposure of these photos beyond their use in research studies.

In summary, the aims of the current study were twofold. First, we compared how individuals process generic and personally relevant facial expressions of anger, sadness, and happiness relative to neutral facial expressions across measures of selective attention and cognitive inhibition. Differences in selective attention allocated to emotional stimuli can be estimated using a modified version of the spatial cueing task (Stormark et al., 1995; Ellenbogen et al., 2002) that measures the latency to respond to a neutral target following either a valid or invalid cue. Cues are valid when presented in the same hemifield as the target, and invalid when presented to the hemifield contralateral to the target. The valid vs. invalid manipulation permits the differentiation in reaction time between the engagement and disengagement of spatial attention (Posner, 1978). Importantly, the adapted modified procedure measures how efficiently one disengages their attention from emotional stimuli. There is increasing evidence that early automatic processing of emotional stimuli relates to different functions (i.e., fear processing) and brain circuits than slower conscious processing (Morris et al., 1999; Ellenbogen et al., 2006). Therefore, in the current study, generic and personally relevant stimuli were presented either briefly followed by a mask to assess automatic processing with limited conscious awareness, or at a long exposure duration allowing full conscious processing. Cognitive inhibition was assessed using the negative affective priming (NAP) task (Goeleven et al., 2006). Cognitive inhibition is defined as the ability to suppress interferences from distracting emotional information in one’s environment. Because working memory has a limited capacity, its efficient functioning depends on inhibitory processes that limit the access of irrelevant information into consciousness. If one’s cognitive inhibition capacity is weakened, too much irrelevant information enters into working memory, impeding our ability to respond flexibly and adapt our behavior and emotional responses to the environment (Hasher et al., 1999). Using the NAP task to measure cognitive inhibition, participants are instructed to respond on multiple trials to a target emotional stimulus (i.e., angry, sad, and happy faces) while simultaneously ignoring or inhibiting an emotional (task-irrelevant) distractor. The NAP effect refers to a participant’s trial response time for the target emotional stimulus (e.g., sad faces) when the same stimulus was the inhibited distractor on the previous trial (i.e., NAP trials; see Figure 1). Delays in responding on NAP trials represents increased cognitive inhibition capacity, while faster responding represents poorer inhibition of emotional distractors (Joormann, 2010).

Second, we examined whether information processing of an intimate partner’s face is associated with relationship quality (i.e., conflict and support), as a means of providing evidence of the validity for this methodology. Given that the personally relevant pictures depicted participants’ intimate partners, we chose to examine whether attention and inhibition of these stimuli predicted the quality of the romantic relationship. Further, previous studies have demonstrated that conflict and support in a romantic relationship predict satisfaction with the relationship (Cramer, 2004; Campbell et al., 2005), depression symptoms (Mackinnon et al., 2012; Marchand-Reilly, 2012), and overall well-being (Gere and Schimmack, 2011). Therefore, given the importance of these constructs, the present study focused on conflict and support within the romantic relationship.

We hypothesized that the personally relevant stimuli, relative to the generic stimuli, will elicit greater selective attention and inhibition overall. Because threat is linked to fear systems associated with early automatic processing (Morris et al., 1999), we expected that differences in threat between generic and personally relevant stimuli would be minimal with perhaps a lesser effect for personally relevant than generic stimuli for early automatic processing. However, as sad affect may be more meaningful in a personally relevant context (i.e., empathy and potential signals of problems within the relationship) compared to generic stimuli (i.e., empathy alone), we expected the greatest difference in information processing for sad personally relevant faces relative to the sad faces of strangers. Lastly, we predicted that greater selective attention and less inhibition toward personally relevant sad faces, relative to generic sad faces, would be associated with more conflict and less support within romantic relationships. We also examined sex differences but had no *apriori* hypotheses given that there are few studies of the topic in the research area.

## Materials and Methods

### Participants

Fifty participants (25 females), aged between 18 and 29 years old (*M* = 22.9 years, *SD* = 3.1 years), were recruited in the Montréal, Québec area through the Concordia University participant pool website, as well as local and online advertisements. Participants and their partners were required to be in a romantic relationship for a minimum of 6 months at the time of testing. Twenty-eight percent of participants reported being in their relationship between 6 months and 1 year, 46% between 1 and 3 years, 20% between 3 and 6 years and 6% reported 6 years or more. Fifty-two percent of participants reported living with their partner. Exclusion criteria include any past or present diagnosis of schizophrenia, bipolar disorder, pervasive developmental disorder, or current substance abuse/dependence in either the participant or their partner, visual impairment, major medical illness within the past 3 weeks, or the use of a psychotropic medication at the time of the study.

### Measures

#### The Structured Clinical Interview for DSM-IV (SCID-I; First et al., 2002)

The SCID-I, a valid and reliable diagnostic instrument (e.g., Zanarini and Frankenburg, 2001), was used to assess parents’ current and past mental health.

#### Quality of Relationships Inventory

The Quality of Relationships Inventory (QRI; Pierce et al., 1991) is a self-report measure used to assess the supportive (i.e., the extent to which the participant perceives availability of support) and conflictual (i.e., perceived conflict within the relationship) aspects within close relationships. This inventory included 25 items rated on a 4-point Likert scale ranging from “not at all” to “always or very much.” In this study, we use the support and conflict scales, but not the depth scale (the importance of the relationship to the participant). Sums of each subscale were calculated with higher scores signifying greater perception of support and conflict. Internal consistency for the current sample was α = 0.91 (conflict) and α = 0.77 (support).

#### The Beck Depression Inventory-II

The Beck Depression Inventory–second edition (BDI-II; Beck et al., 1996) was used to assess the presence of depressive symptoms over the past 2 weeks. Higher scores on the 21-item inventory are indicative of greater depressive symptoms. The internal consistency (α) coefficient for the current sample was 0.80.

### Materials

#### Personally Relevant and Generic Stimuli

The Facial Action Coding System (FACS; Ekman et al., 2002) was used to train the partner of the participant to make four facial expressions: neutral, angry, sad and happy. Each component of the facial expressions was shown and described in detail (e.g., bearing of the teeth to portray anger). Partners were encouraged to use mental imagery to help produce the desired facial expressions and also had a mirror available for practice. If more guidance was needed, the experimenter would model the different facial expressions for each emotion. The pictures were taken using a Canon PowerShot A590IS 8.0 megapixel digital camera mounted on a tripod. Seven to ten pictures were taken for each emotion. Partners wore a gray t-shirt provided to them.

Using criteria from the FACS (Ekman et al., 2002), the five best pictures for each emotion were selected. These pictures were rated by five lab members on a global rating scale from 1 to 10. Pictures were judged on their intensity (degree of emotion conveyed) and their authenticity. The two pictures for each emotion type with the highest average ratings across all raters for both dimensions were selected as stimuli for the experiment. Mean picture ratings across all participants were as follows: neutral (*M* = 7.6, *SD* = 0.64), angry (*M* = 7.2, *SD* = 0.91), sad (*M* = 6.8, *SD* = 0.79), and happy (*M* = 8.1, *SD* = 0.80) stimuli. An intra-class correlation computed across all raters indicated high inter-rater reliability (ICC = 0.995).

Personally relevant pictures were processed using Adobe Photoshop CS4 editing software. Pictures were reduced to 60 by 90 pixels, the background was made uniform, and the brightness and hue were adjusted to match the generic stimuli. With regards to the generic pictures, a set of 12 (6 male) Caucasian faces each displaying a neutral, angry, sad and happy expression were selected from the MacArthur Network Face Stimuli Set (NimStim stimulus set; Tottenham et al., 2009).

### Modified Spatial Cueing Task

A modified version (Ellenbogen et al., 2002) of the spatial cueing visual attention paradigm (Posner, 1980) was used to assess attentional engagement and disengagement toward generic and personally relevant stimuli. Participants fixated on a centrally located “+” sign on a black background, which was flanked on both sides by a gray rectangle (3.7 cm × 3.2 cm). The center of each rectangle was 2.2° of visual angle from the fixation point. A small black dot (i.e., target) appeared in either of the gray rectangles. Preceding each target presentation, a cue appeared in one of the rectangles. The cue was indicative of where the target would appear. The cues were pictures of faces displaying one of the four emotion types (neutral, angry, sad, or happy), and were either generic or personally relevant. Participants were asked to respond as quickly as possible once the target appeared.

There were 384 trials (192 personally relevant), divided into 16 blocks (4 blocks for each emotion type) of 24 trials, and 20 additional “catch” trials presented across blocks, where the target did not appear following the presentation of the cue. Catch trials (not included in statistical analyses) were used to prevent participants from developing an automatic response set due to the fixed cue-target intervals in this experiment. On valid or attentional engagement trials (288), the cue and target appeared in the same hemifield. On invalid or disengagement trials (96), the cue and target appeared in opposite hemifields. The stimulus onset asynchrony (the interval between the onset of the cue and onset of the target) for valid and invalid trials was 830 ms, and the interval between trials (from the offset of the target to next cue onset) was 2,200 ms. Cues were presented for 750 ms or 17 ms followed by a masking stimulus. For masked trials, the mask was presented for 183 ms immediately at the offset of the cue. At 80 ms following the offset of the cue or mask, targets were presented for 600 ms. The masks were made by digitally cutting pictures into small pieces and randomly reassembling them.

Within each block, the picture cues were of the same emotion type to avoid affective carryover effects, where the emotion type of the cue on one trial influences the response on the subsequent trial. The order of blocks and hemifield of stimulus presentation were varied randomly across subjects, except that two blocks of the same picture category were never consecutive. Validly and invalidly cued targets represented 75 and 25% of all trials, respectively, a ratio shown to be effective in cueing attention (Posner, 1978). Reaction times (RT) less than 150 ms and more than 850 ms were excluded from the analyses.

An index of disengagement score was computed by subtracting RTs of neutral picture cues from RTs of emotional picture cues for invalid trials.

An index of engagement was computed by subtracting RTs of emotional picture cues from RTs of neutral picture cues for valid trials. For both indices, positive scores indicate greater selective attention to the emotional cue (i.e., faster engagement to, or slower disengagement from, an emotional face) whereas negative scores signal attentional avoidance (i.e., slower engagement to, and faster disengagement from, an emotional face).

### Negative Affective Priming Task

Derived from the original negative priming paradigm (Tipper and Cranston, 1985) and its later adaptation (Goeleven et al., 2006; Taylor et al., 2011), a computerized cognitive task was designed to assess participants’ ability to inhibit generic and personally relevant facial stimuli depicting angry, sad and happy emotional expressions. Participants were instructed to use a two-key response box to identify, as quickly as possible, whether the stimulus presented in a white frame (target) depicted a positive or a negative facial expression, while ignoring the stimulus presented in a black frame (distractor). Target and distractor stimuli were presented simultaneously at either the top or the bottom of the screen.

Specifically, the negative affective priming (NAP) task consisted of fixed consecutive pairs of “prime” and “test” trials (see Figure 1). Prime trials always preceded the test trials. In the negative priming condition, the emotional expression of the target picture during the test trial was the same as the emotional expression of the previously ignored distractor in the prime trial. In the control condition, the emotional expression of the target picture during the test trial was unrelated to the emotional expression of the previously ignored picture in the prime trial. Importantly, the pictures presented during the test trial of the negative priming and control conditions were identical; the conditions only differed in the pictures presented in the prime trials. In order to counterbalance the type of emotional stimulus used as targets in prime and test trials, as well as distractors in test trials, two sequences of negative priming and control manipulations were used. Trials were also counterbalanced for the spatial location of the pictures. The design of the NAP task was identical for both the personally relevant and generic stimuli, and differed only in the number of distinct actors conveying the emotional expressions.

One hundred and ninety-two stimulus presentations were paired (priming and test trials) into 96 trials (48 negative priming trials and 48 control trials), appearing in random sequence. Participants were presented with an equal number of paired trials for each emotional expression (32 angry, 32 sad, 32 happy), half of which consisted of personally relevant pictures. Trial sequences (priming followed by a test trial) used either personally relevant or generic pictures; they were never mixed. Pictures remained on the screen until a response was provided or for a maximum of 7,500 ms. Each trial was separated by an inter-stimulus interval of 1,000 ms during which time a centered fixation cross would appear.

An index of inhibition score was computed for each emotional expression by subtracting mean RT on matched control test trials from mean RT on matched negative priming test trials. A positive index value indicates strong inhibition, meaning that the emotional expression of the distractor presented during the prime trial led to a slower RT during the test trial of the same emotion. Conversely, a negative index value indicates reduced/poor inhibition because the distractor presented during the prime trial prompted a faster RT during the test trial. Reaction times below 150 ms and above 4,000 ms, as well as incorrect responses to test trials, were excluded from the analyses.

### Procedure

Following completion of the screening protocol, including a structured interview assessing past and current mental health, participants and their intimate partners were scheduled for separate sessions. First, partners took part in a photography session to obtain the personally relevant stimuli (as described above). Approximately 1 week later, participants completed the aforementioned information processing tasks in random order, as well as an emotion-modulated acoustic startle task. The electromyography startle data were of questionable quality, and were thus excluded from the present study. Participants used a chin rest throughout the tasks to maintain a distance of 57 cm from the computer monitor. All tasks were performed on an IBM PC computer, with a 17 in. NEC color monitor, which were programmed using the STIM Stimulus Presentation System software (version 7.584) developed by the James Long Company (Caroga Lake, NY). Following completion of the tasks, the questionnaires were completed. Participants and their partners were debriefed and remunerated for the time spent in the laboratory. All procedures were approved by the Human Research Ethics Committee of Concordia University (Montréal, Québec), and participants and their partners provided written informed consent.

### Data Analysis

Power analyses for the within-subject comparison of personally relevant and generic stimuli were calculated using *G^∗^Power 3.1* (Faul et al., 2007), using an effect size of 0.16 (Cohen’s *f*; small-medium effect size). The effect size of 0.16 was determined from a previous study comparing inhibition scores (happy, sad, angry) for personally relevant vs. generic stimuli (Iacono et al., 2015). The analysis indicated that 50 participants would provide sufficient power (0.80, *p* < 0.05) to test the study’s primary hypothesis. Data were screened for outliers (±3 standard deviations from the mean) and distributional anomalies. Prior to testing the hypotheses, tests of validity for each task were conducted. The data were then analyzed to determine the effect of personal relevance for each task. For the modified spatial cueing task, a Sex × Relevance (generic, personally relevant) × Emotion (neutral, happy, sad, angry) × Exposure (750 ms, masked) repeated measures ANOVA was performed on engagement and disengagement scores separately. For the NAP task, a Sex × Relevance × Emotion repeated measures ANOVA was conducted on inhibition scores.

Finally, a series of hierarchical multiple regressions predicting relationship quality (i.e., support and conflict) from indices of social information processing were conducted. For the spatial cueing data, the predictor variables were entered in three steps: (1) BDI scores (to control for depression) and participant’s sex, (2) centered disengagement scores from generic and personally relevant stimuli, and (3) a Sex × Centered Personally Relevant Stimuli. Models were ran separately for each emotion type and repeated for both 750 ms and masked cue presentations. Similar hierarchical multiple regressions were performed on engagement scores from valid trials of the spatial cueing task and inhibition scores from the NAP. Significant interactions were followed-up using a test of simple slopes (Aiken and West, 1991).

## Results

### Spatial Cueing Task

To test the validity of the spatial cueing task, RT data were analyzed with a Picture relevance × Cue Validity (valid, invalid) × Emotion × Exposure repeated measures ANOVA. As expected, a main effect of cue validity was found for trials with the 750 ms cue presentation, [*F*_(1,49)_ = 17.790, *p* = 0.000, η^2^ = 0.266] and masked cues [*F*_(1,46)_ = 119.550, *p* = 0.000, η^2^ = 0.709]. Specifically, participants had slower RT for invalid relative to valid trials for both 750 ms and masked presentations, supporting the validity of the spatial cueing paradigm. For the 750 ms presentation, mean RT was 320 ms (*SD* = 50 ms) for invalid trials and 306 ms (*SD* = 48 ms) for valid trials. For the masked presentation, mean RT was 318 ms (*SD* = 47 ms) for invalid trials and 284 ms (*SD* = 40 ms) for valid trials.

#### Disengagement (Invalid) Trials

To examine the effect of picture relevance on attentional disengagement, a Sex × Picture Relevance × Emotion × Exposure repeated measures ANOVA was conducted using the disengagement index scores. A significant main effect of relevance was observed [*F*_(1,48)_ = 4.257, *p* = 0.045, η^2^ = 0.081], with participants being faster to shift their attention away from personally relevant stimuli than generic stimuli (see Table 1).

**TABLE 1:** Mean index scores (emotion minus neutral) and standard deviations (SD) for the processing of generic and personally relevant stimuli during engagement and disengagement trials of the spatial cueing task across all emotion types.

	**Male**	**Female**	**Total**
	**Mean (*SD*)**	**Mean (*SD*)**	**Mean (*SD*)**
**Engagement Trials**			
750 ms			
Angry Generic	−1.70(20.82)	−13.10(35.03)	−7.40(29.09)
Angry PR	6.10(30.89)	−9.90(32.62)	−1.90(32.47)
Sad Generic	−6.00(28.83)	3.60(37.58)	−1.20(33.50)
Sad PR	12.40(23.14)	−0.60(23.83)	5.90(24.15)
Happy Generic	−0.20(28.97)	−4.80(39.03)	−2.50(34.10)
Happy PR	8.80(26.86)	7.10(25.31)	7.90(25.84)
Masked			
Angry Generic	0.90(20.27)	−6.00(34.56)	−3.50(28.32)
Angry PR	−5.70(16.86)	−4.50(25.88)	−5.10(21.62)
Sad Generic	6.70(31.37)	5.60(36.70)	6.20(33.80)
Sad PR	−6.60(20.56)	2.90(37.91)	−1.90(30.56)
Happy Generic	7.50(20.55)	5.90(29.42)	6.70(25.13)
Happy PR	4.10(20.03)	2.90(32.96)	3.50(27.00)
**Disengagement Trials**			
750 ms			
Angry Generic	6.00(39.48)	10.20(49.96)	8.10(44.20)
Angry PR	5.20(41.91)	−3.10(56.66)	1.00(49.50)
Sad Generic	3.20(34.73)	−2.40(58.18)	.40(47.51)
Sad PR	2.40(31.77)	−11.20(44.70)	−4.40(38.99)
Happy Generic	−5.40(42.97)	2.70(50.68)	−4.10(46.52)
Happy PR	3.20(46.72)	−7.10(56.56)	−1.90(51.61)
Masked			
Angry Generic	5.30(34.22)	12.40(59.59)	8.80(48.23)
Angry PR	−14.40(43.85)	5.60(42.45)	−4.40(43.89)
Sad Generic	4.60(32.73)	8.20(56.90)	6.40(45.98)
Sad PR	−21.60(38.90)	0.80(51.31)	−10.40(46.46)
Happy Generic	8.90(37.37)	11.60(58.36)	10.30(48.52)
Happy PR	−13.90(49.93)	1.10(44.83)	−6.40(47.57)

*Means and standard deviations are presented in milliseconds (ms).*

#### Engagement (Valid) Trials

**FIGURE 2:**
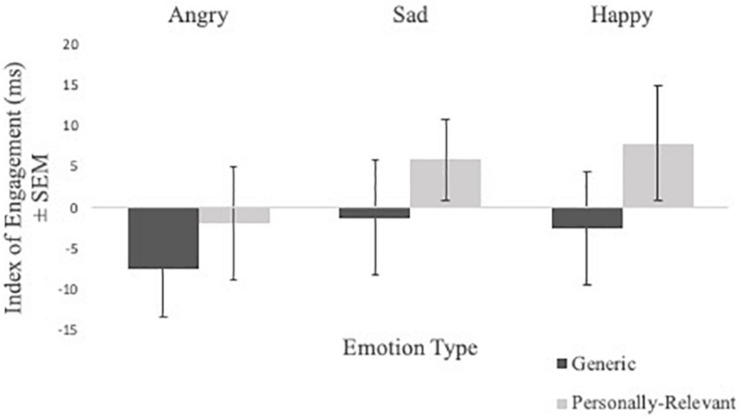
Comparisons of mean index scores of engagement (i.e., RT valid trials with emotional stimuli—RT valid trials with neutral stimuli) of angry, sad, and happy facial expressions for generic and personally relevant stimuli for 750 ms presentation time. Relative to generic pictures of facial expressions of emotion, participants shifted attention more quickly toward facial expressions of their intimate partner when presented for 750 ms, but not masked presentations. Error bars represent standard errors.

Engagement index scores from the valid trials of the spatial cueing task were subject to a Sex × Picture Relevance × Emotion Type × Exposure repeated measures ANOVA. A significant Picture Relevance × Exposure interaction was observed [*F*_(1,48)_ = 4.376, *p* = 0.042, η^2^ = 0.084]. To follow-up, a Picture Relevance × Emotion Type repeated measures ANOVA was conducted separately for 750 ms exposure and masked trials. For 750 ms exposure trials, a main effect of relevance was observed [*F*_(1,48)_ = 4.090, *p* = 0.049, η^2^ = 0.079], where participants were quicker to shift toward personally relevant faces, relative to generic stimuli (see Figure 2). No significant findings were found for masked trials.

In sum, personally relevant stimuli, regardless of whether the facial expression was angry, sad, or happy, elicited attentional avoidance on disengagement trials (on both 750 ms and masked disengagement trials) and rapid shifts of attention toward stimuli (on 750 ms engagement trials) relative to trials with generic facial expressions of emotion. See Table 1 for means and standard deviations of the spatial cueing data.

### Negative Affective Priming Task

To verify the validity of the NAP task, a Priming Condition (negative priming, control) × Emotion Type × Picture Relevance repeated measures ANOVA was conducted on RT data. A main effect for priming condition [*F*_(1,56)_ = 13.294, *p* = 0.001, η^2^ = 0.192] was observed, with participants demonstrating overall significantly slower RTs on negative priming trials (*M* = 954 ms, *SD* = 272 ms) compared to control trials (*M* = 916 ms, *SD* = 268 ms), supporting the validity of the NAP paradigm.

**FIGURE 3:**
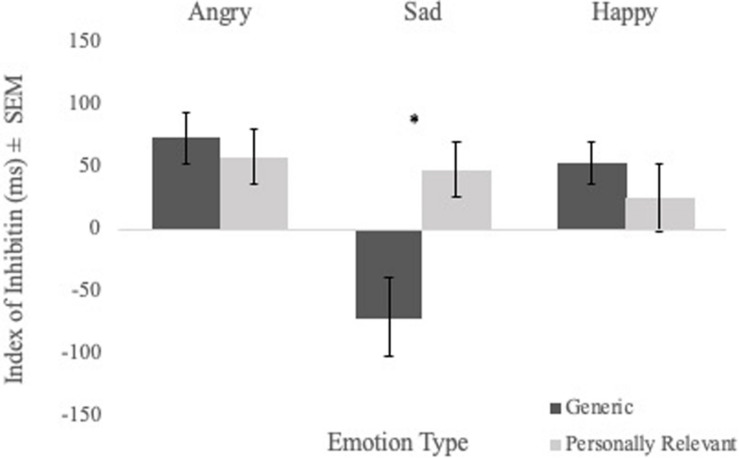
Comparisons of mean inhibition index scores (i.e., mean RT on control trials—mean RT on priming trials) of angry, sad, and happy facial expressions for generic and personally relevant stimuli. Relative to generic pictures of facial expressions of sadness, participants exhibited increased inhibition of the sad face of their intimate partner. *Note.* Error bars represent standard errors. *p < 0.05.

To examine the effect of picture relevance on inhibition, a Sex × Picture Relevance × Emotion Type repeated measures ANOVA of inhibition index scores was performed. No significant main effect of picture relevance was found. However, the Picture Relevance × Emotion Type interaction was significant [*F*_(1,56)_ = 5.970, *p* = 0.003, η^2^ = 0.096]. Follow-up analyses revealed a main effect of relevance for sad faces [*F*_(1,56)_ = 12.441, *p* = 0.001, η^2^ = 0.182], with participants demonstrating strong inhibition of personally relevant sad stimuli but poor inhibition of generic sad stimuli (see Figure 3). There were no main effects for angry or happy faces.

### Social Information Processing of Personally Relevant Stimuli and Relationship Quality

**FIGURE 4:**
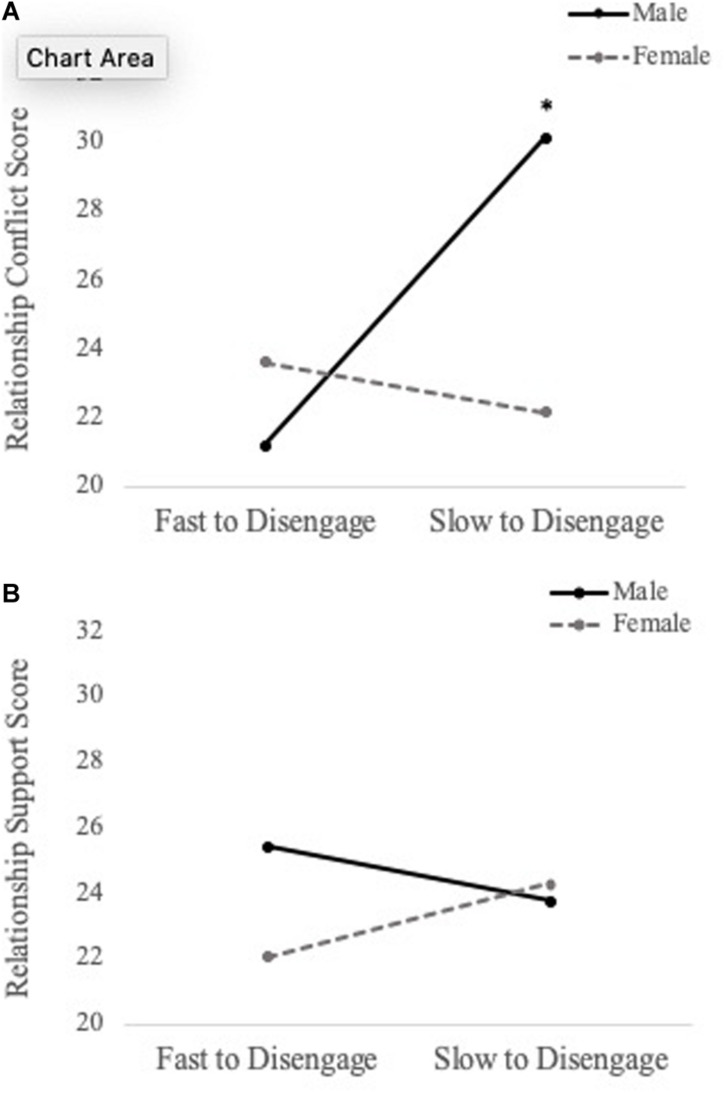
Simple slopes analyses demonstrating the relation between attentional disengagement of personally relevant sad faces (750 ms) and level of conflict **(A)** and support **(B)** in romantic relationship for males and females. Difficulties shifting attention away (increased selective attention) from the sad face of their intimate partner was associated with greater levels of conflict for male participants, but not female participants. Similar findings were observed for relationship support, but neither slope was significantly different than zero. *p < 0.05.

To examine whether social information processing of personally relevant stimuli predicts relationship quality (i.e., conflict and support), a series of hierarchical multiple regressions were performed. For 750 ms cue presentation time, we found a main effect of disengagement from generic sad faces when predicting levels of conflict (*b* = -49.116, *SE* = 18.324, *t* = -2.680, *p* = 0.010), such that fast disengagement from generic sad faces predicted increased conflict. Additionally, there was a significant Sex × Personally Relevant Sad Stimuli interaction (*b* = -53.890, *SE* = 23.439, *t* = -2.299, *p* = 0.026). With respect to the overall effect size, the model was found to explain 38.7% of the variance in relationship conflict scores. Simple slopes analyses revealed that the slope for male participants was significantly different than zero [*b* = 85.715, *t*(45) = 2.107, *p* = 0.04]. Specifically, increased attention toward personally relevant sad faces was associated with greater levels of conflict for males, but not females (see Figure 4A).

The same hierarchical regression models were conducted to predict relationship support. For 750 ms cue presentation time, there was a main effect of disengagement from generic sad faces when predicting levels of support (*b* = 22.324, *SE* = 10.883, *t* = 2.051, *p* = 0.046), such that fast disengagement from generic sad faces predicted less support. Moreover, the Sex × Personally Relevant Sad Faces interaction was a significant predictor of support in the relationship (*b* = 22.324, *SE* = 10.883, *t* = 2.051, *p* = 0.046). With respect to the overall effect size, the model was found to explain 26.8% of the variance in relationship support scores. Despite the statistically significant interaction, simple slope analyses revealed both the slopes for male and female participants were not significantly different from zero (see Figure 4B). The above results are summarized in Table 2 and the Pearson correlations between these study variables are presented in Table 3.

**TABLE 2:** Multiple regression results for predictors of the level of conflict and support in intimate relationships for the invalid sad face trials of the spatial cueing task (750-ms presentation time).

		**Unstandardized coefficient**		**Standardized coefficients**				
		***b***	***SE***	***B***	***t***	***p***	**Δ*R*^2^**	***R*^2^**
**Predicting conflict**								
Step 1	Constant	19.84	1.30	–	15.21	0.00		0.263
	BDI score	0.50	0.10	0.56	4.83	0.00		
	Sex of participant	–2.08	0.87	–0.28	–2.41	0.02		
Step 2	PR sad faces	35.93	23.41	0.19	1.54	0.13	0.066*	0.329
	Generic sad faces	–49.12	18.32	–0.31	–2.68	0.01		
Step 3	Sex × PR sad faces	–53.89	23.44	–0.27	–2.30	0.03	0.058*	0.387
**Predicting support**								
Step 1	Constant	25.37	0.61	–	41.90	0.00		0.109
	BDI score	–0.15	0.05	–0.42	–3.31	0.00		
	Sex of participant	–0.33	0.40	–0.10	–0.81	0.42		
Step 2	PR sad faces	1.28	10.87	0.02	0.12	0.01	0.107*	0.216
	Generic sad faces	25.76	8.51	0.38	3.03	0.00		
Step 3	Sex × PR sad faces	22.32	10.88	0.27	2.05	0.04	0.052*	0.268

*PR, personally relevant; ^∗^p < 0.05.*

**TABLE 3:** Pearson correlation coefficients for study variables.

**Variable**	**1**	**2**	**3**	**4**	**5**	**6**	**7**	**8**	**9**	**10**	**11**	**12**	**13**	**14**	**15**	**16**	**17**	**18**	**19**	**20**
Sex		-0.13	0.22	0.18	–0.05	0.06	0.09	0.18	–0.07	–0.04	–0.23	–0.24	0.19	–0.14	0.25	0.27	0.09	0.02	–0.03	–0.16
BDI score			0.46*	−0.36*	0.29*	0.20	–0.06	0.01	0.08	0.10	0.21	0.23	–0.17	–0.05	–0.18	–0.13	–0.12	–0.26	0.03	0.04
QRI Conflict				−0.67*	0.05	–0.16	0.01	0.15	–0.08	–0.19	–0.11	0.01	0.03	0.02	0.08	–0.04	–0.12	–0.13	0.15	0.10
QRI Support					–0.10	0.29*	0.16	0.12	0.17	0.07	0.00	–0.14	0.16	0.09	0.03	0.10	0.06	0.07	–0.10	0.06
**Disengagement Trials**																				
**750 ms**																				
Generic angry						0.40*	0.14	–0.04	0.32	0.39*	0.08	0.16	–0.25	–0.20	–0.14	–0.06	0.00	–0.08	–0.06	–0.09
Generic sad							–0.03	–0.00	0.15	0.14	0.04	0.17	–0.18	–0.17	–0.05	0.07	–0.02	–0.14	–0.16	–0.10
PR angry								0.53*	0.33*	0.12	0.20	0.01	–0.09	–0.15	–0.02	–0.01	–0.26	–0.27	–0.23	–0.08
PR sad									0.20	0.05	–0.20	–0.18	0.13	–0.11	–0.02	0.11	–0.28	–0.39	–0.11	–0.18
**Masked**																				
Generic angry										0.48*	0.03	–0.01	–0.00	–0.18	−0.36*	–0.19	–0.02	0.14	–0.31	0.05
Generic sad											0.10	0.24	0.08	–0.23	–0.04	–0.24	0.22	0.07	−0.32*	–0.28
PR angry												0.38*	–0.16	0.13	–0.15	–0.07	–0.01	–0.10	–0.22	0.01
PR sad													–0.21	−0.29*	–0.26	–0.22	0.06	–0.22	–0.27	–0.25
**Engagement Trials**																				
**750 ms**																				
Generic angry														0.61*	0.39*	0.16	0.01	0.10	0.00	0.08
Generic sad															0.42*	0.06	–0.12	0.01	0.23	0.42*
PR angry																0.17	–0.10	–0.22	0.19	–0.17
PR sad																	0.08	0.05	0.15	0.18
**Masked**																				
Generic angry																		0.54*	0.15	0.08
Generic sad																			0.04	0.29*
PR angry																				0.43*
PR sad																				

*PR, personally relevant; ^∗^p < 0.05.*

We examined whether disengagement from the other emotional stimuli predicted relationship quality. The results of these analyses were not statistically significant, suggesting the findings were specific to sad facial expressions (data not shown). In addition, there were no significant findings for masked presentation trials or when using engagement scores of the spatial cueing task (data not shown). Similar hierarchical regressions were performed for the negative priming and task, but did not yield statistically significant results (data not shown).

## Discussion

The purpose of the present study was twofold. First, we compared selective attention and inhibition of emotional faces using generic and personally relevant stimuli. Second, with the goal of providing evidence of the predictive validity of this methodology, we assessed whether the information processing of personally relevant stimuli predicted relationship quality. A number of key findings emerged. Consistent with predictions, the present study provides evidence that personally relevant facial expressions of emotion, using pictures of participants’ intimate partners, are processed differently than pictures of strangers’ facial expressions taken from generic databases. Relative to generic pictures, participants shifted attention more quickly toward facial expressions of their intimate partner, regardless of valence, when presented with full conscious awareness (750 ms). Attentional disengagement, in contrast, was faster (indicative of attentional avoidance) when shifting attention away from the facial expressions of their intimate partner relative to generic pictures for both the masked and 750 ms presentations. For cognitive inhibition, the findings were emotion-specific, as predicted. While participants were unable to inhibit the processing of sad generic faces, similar to previous studies using this paradigm (Taylor et al., 2011; Ellenbogen et al., 2013), they showed strong inhibition of personally relevant sad facial expressions. Finally, partly consistent with our hypothesis, difficulties disengaging attention from personally relevant sad faces predicted increased conflict in their relationship among male but not female participants. A similar pattern of results was found for relationship support, although the effect was weaker than ratings of conflict. Unexpectedly, the efficiency to disengage attention from generic sad faces also predicted relationship quality, such that fast disengagement (avoidance) from generic sad faces predicted more conflict and less support in participants’ current relationship.

Personally relevant stimuli elicited both increased selective attention and avoidance relative to generic stimuli depending on the type of trial and exposure duration. The selective attention effect, occurring only when shifting attention toward the emotional cue, supports the view that personally relevant stimuli attract attention and are more salient than generic stimuli (Wingenfeld et al., 2006; Fridrici et al., 2013). The fact that this effect was evident across all facial expressions of emotion suggests that it is likely due to familiarity and unrelated to the expression of a specific emotion (Bartels and Zeki, 2000; Leibenluft et al., 2004; Viskontas et al., 2009). In contrast to increased selective attention, the observed attentional avoidance of personally relevant stimuli might represent an attempt to regulate negative affect caused by their presentation (Ellenbogen et al., 2002; Cisler and Koster, 2010). In the present study, the fast disengagement from the face of one’s intimate partner might reflect enhanced processing of the familiar vs. generic faces, enabling participants to engage in fast task-consistent shifts away from emotional faces. Moreover, previous research has shown that threatening stimuli are less attended to following mere exposure (Young and Claypool, 2010). Therefore, it is also possible that previous exposure to personally relevant facial expressions may underlie the participants’ ability to quickly disengage from their intimate partner’s face compared to that of a stranger. On the other hand, generic stimuli might capture attention more readily, and thus slow down disengagement, via the activation of threat-related circuits associated with fast automatic processing (Morris et al., 1999). Given that the rapid shifts of attention toward and away from personally relevant faces were consistent with task instructions (i.e., to respond to a target that is correctly cued in engagement trials, and to shift attention away when the target is incorrectly cued in a disengagement trial), it is likely that these changes represent adaptive responses to highly salient cues. However, further research is needed to determine the correlates and functional relevance of this fast attentional avoidance to determine whether it is adaptive or maladaptive.

In addition to these general increases in selective attention and avoidance, we found evidence of increased valence-specific (sad) inhibition of personally relevant faces relative to generic ones. In past studies of NAP (Taylor et al., 2011; Ellenbogen et al., 2013), participants were unable to inhibit the processing of distracting generic sad faces. Although the cause of the inhibition failure is unknown, we speculate that difficulties suppressing sad faces may be associated with an automatic empathy response to a distressed stranger, which might impede inhibition. With respect to pictures of a distressed intimate partner, it appears that a different process might be active. Sad affect in an intimate partner would be expected to elicit more complex and personal responses, perhaps signaling relationship problems and eliciting strong emotion. Given the persistent nature of sadness, it might be a more salient indicator of threat to the relationship compared to other negative emotions, such as anger (Ellsworth and Smith, 1988). The effective use of cognitive inhibition is likely an important component in the regulation of sadness. For example, individuals with a history of depression as well as clinically depressed individuals display difficulties inhibiting irrelevant sad stimuli (Joormann, 2004; Goeleven et al., 2006; Joormann and Gotlib, 2010). Thus, the improvement in inhibitory control of personally relevant sad faces may represent an adaptive response to regulate emotions associated with partner distress. In addition, high inhibitory control of these salient stimuli may have positive effects on the regulation of physiological stress reactivity and secretion of the hormone cortisol. We recently found that high chronic stress was associated with poor inhibition of the sad face of one’s intimate partner, and that this change in inhibition predicted an elevated cortisol response at awakening 6 months later (Wong et al., 2020). Taken together, strong inhibitory control of personally relevant sad faces may be related to different indices of adaptive functioning. Future research, however, will be needed to test these predictions.

Importantly, we found evidence that information processing biases predict concurrent relationship quality, although these effects are, in part, related to the sex of the participant and the valence of the stimuli used. Male participants’ slow disengagement from their female partners’ sad face was associated with increased conflict in the relationship. Interestingly, this association was specific to depictions of sadness and was not found in female participants, perhaps indicating greater salience of female emotional facial expressions relative to male facial expressions. Indeed, women express a higher rate of facial emotions and report experiencing more fear, sadness, and joy than men (Allen and Haccoun, 1976; LaFrance and Banaji, 1992). In addition, women were also rated as more emotionally expressive than men in a study of couples using an objective behavioral coding system (Notarius and Johnson, 1982). Thus, facial expressions of emotion displayed by women are perhaps more meaningful and salient in the context of a romantic relationship than similar expressions displayed by men because they occur more frequently and might be more closely tied to problems in the relationship.

Unexpectedly, slow disengagement from generic sad faces presented for 750 ms predicted *increased* support and *less* conflict in the relationship, which is opposite to the findings reported for personally relevant stimuli. Again, differences between generic and personally relevant stimuli are striking, further highlighting the importance of distinguishing between the two types of processing. Perhaps the relationship between general selective attention, as assessed with generic stimuli, and positive relationship quality requires increased selective attention to emotional cues rather than avoidance or suppression, which is known to hinder relationship functioning (Gross and John, 2003; Ben-Naim et al., 2013; Velotti et al., 2016). It is possible that the relationship observed with generic stimuli is tapping into general links between attentional style and interpersonal functioning, whereas personally relevant processing may be tapping into more specific factors regarding the quality of participants’ current relationship.

A key strength of the present research is that it is among a small number studies (Iacono et al., 2015; Wong et al., 2020) to assess information processing using emotional facial expressions of participants’ intimate partners. However, a number of limitations warrant consideration. First, participants were exposed to the face of their partner more frequently during the experiment than the generic stimuli, which used multiple actors. Habituation to the partner’s face may explain some of the differences in the processing of personally relevant and generic stimuli. Future studies may want to match the number of actors used for the generic stimuli with those used for the personally relevant stimuli (e.g., one actor expressing the different emotions). However, the present study intended to mimic how most studies use generic photographic databases and attempted to minimize familiarity effects that occur with multiple presentations of facial emotions from the same actor. Second, considering that the generic stimuli employ trained actors, it is possible that they display more salient or intense depictions of emotion than the personally relevant ones, despite our efforts to train intimate partners to express similar facial emotions. Third, we only measured relationship quality as perceived by the participant. Future studies may benefit from including an objective measure of relationship functioning (e.g., a semi-structured interview) and/or including partners’ reports. Finally, the cross-sectional nature of the assessment of information processing and relationship quality precludes interpretations of direction of effect.

Overall, the present study highlights robust differences in information processing when using generic vs. personally meaningful emotional stimuli, and demonstrates how attentional disengagement biases for sad faces using both types of stimuli relate to current relationship functioning. Future research should attempt to replicate these findings with a larger sample and test out directional hypotheses using a prospective study design. If reliability and validity are shown in future studies, personally relevant social information tasks could open new areas of social and neurobiological research. Moreover, the possibility of using low-cost information processing tasks to predict relationship quality could have important clinical implications, given the strong links between poor interpersonal functioning and health (Holt-Lunstad et al., 2008).

## Data Availability Statement

The datasets generated for this study are available on request to the corresponding author.

## Ethics Statement

The studies involving human participants were reviewed and approved by the Human Research Ethics Committee of Concordia University. The patients/participants provided their written informed consent to participate in this study.

## Author Contributions

LS ran the statistical analyses. LS and VT produced the first draft of the manuscript. ME designed the study and edited the manuscript. All authors contributed to the article and approved the submitted version.

## Conflict of Interest

The authors declare that the research was conducted in the absence of any commercial or financial relationships that could be construed as a potential conflict of interest.
